# Aetiology of acute febrile illness among children attending a tertiary hospital in southern Ethiopia

**DOI:** 10.1186/s12879-020-05635-x

**Published:** 2020-11-30

**Authors:** Techalew Shimelis, Birkneh Tilahun Tadesse, Fitsum W/Gebriel, John A. Crump, Gill Schierhout, Sabine Dittrich, John M Kaldor, Susana Vaz Nery

**Affiliations:** 1 Kirby Institute, University of New South Wales, Sydney, Australia; 2 College of Medicine and Health Sciences, Hawassa University, Hawassa, Ethiopia; 3 Centre for International Health, University of Otago, Dunedin, New Zealand; 4 George Institute for Global Health, Sydney, Australia; 5 Foundation for Innovative New Diagnostics, Geneva, Switzerland; 6 Nuffield Department of Medicine, University of Oxford, Oxford, UK

**Keywords:** Malaria, Aetiologies, Acute febrile illness, Bacteraemia, Urinary tract infection

## Abstract

**Background:**

The diagnosis of non-malarial aetiologies, which now represent the majority of febrile illnesses, has remained problematic in settings with limited laboratory capacity. We aimed to describe common aetiologies of acute febrile illness among children in a setting where malaria transmission has declined.

**Methods:**

A prospective cross-sectional study was conducted among children aged at least 2 months and under 13 years presenting with fever (temperature of ≥37.5 °C or a history of fever in the past 48 h) to Hawassa Comprehensive Specialized Hospital, southern Ethiopia, from May 2018 through February 2019. Clinical and demographic data were gathered for consecutive participants, and malaria microscopy, HIV testing, and blood and urine cultures were performed regardless of clinical presentation. Additionally, stool analyses (culture and rotavirus/adenovirus RDT) and throat swab for group A *Streptococcus* (GAS) and urine *Streptococcus pneumoniae* were performed by RDTs for children with specific conditions. The antimicrobial susceptibility of bacterial isolates was determined using disc diffusion method.

**Results:**

During the study period 433 children were recruited, median age 20 months (range, 2 months – 12 years) and 178 (41.1%) female. Malaria was diagnosed in 14 (3.2%) of 431 children, and 3 (0.7%) had HIV infection. Bacteraemia or fungaemia was detected in 27 (6.4%) of 421 blood cultures, with *Staphylococcus aureus* isolated in 16 (3.8%). Urinary tract infections (UTIs) were detected in 74 (18.4%) of 402, with *Escherichia coli* isolated in 37 (9.2%). Among 56 children whose stool specimens were tested, 14 (25%) were positive for rotavirus, 1 (1.8%) for *Salmonella* Paratyphi A, and 1 (1.8%) for *Shigella dysenteriae*. Among those with respiratory symptoms, a throat swab test for GAS and urine test for *S. pneumoniae* were positive in 28 (15.8%) of 177 and 31 (17.0%) of 182, respectively. No test was positive for a pathogen in 266 (61.4%) of 433 participants. Bacterial isolates were frequently resistant to ampicillin, trimethoprim-sulfamethoxazole, tetracycline, and amoxicillin and clavulanic acid.

**Conclusion:**

Our results showed low proportions of malaria and bacteraemia among febrile children. In contrast, the frequent detection of UTI emphasize the need to support enhanced diagnostic capacity to ensure appropriate antimicrobial intervention.

**Supplementary Information:**

The online version contains supplementary material available at 10.1186/s12879-020-05635-x.

## Background

Despite substantial global progress in reducing child mortality, the annual deaths of more than 6 million children aged ≤15 years, 85% in under-5 years old, warrants intensified efforts, particularly in sub-Saharan Africa where the highest mortality ratios occur [1]. More than half of child mortality is directly attributable to infectious diseases of bacterial, viral, parasitic, and fungal origin [2, 3], with fever a key presenting symptom and often the main reason for seeking health care [4].

In many tropical countries, malaria has long been a major cause of mortality in children, and typically presented with fever as a primary symptom [5]. Improved access to diagnosis, particularly with the introduction of sensitive and specific rapid diagnostic tests (RDTs) [6, 7], has allowed health workers to better manage malaria, as well as rule it out with a negative test. However, the diagnosis of the causes of non-malaria febrile illness has remained problematic in resource-constrained countries where laboratory facilities are limited or non-existent [5]. In such settings, management guidelines rely heavily on clinical diagnosis even though fever aetiologies can be difficult to distinguish clinically [3]. Empiric patient management can lead to diseases being undertreated, or treated with unnecessary drugs. Overuse or inappropriate use of antimicrobial agents is also recognised as a major driver of drug resistance, an ever-growing threat to global health [8], which has led to several infections becoming harder to treat as drugs lose their effectiveness against pathogens [9].

The challenge of managing febrile illnesses in the absence of adequate laboratory support demand systematic investigation to guide improvement of management approaches and strengthen disease control efforts at a local level. The World Health Organization (WHO) recognises the importance of studying fever aetiologies in various settings, age groups, and level of care [3], but there have been relatively few such investigations in the countries most affected. Available studies from African countries have found that most children presenting with fever had acute respiratory or gastrointestinal infections which were mainly attributed to viral pathogens [10, 11], and thus not amenable to antimicrobial treatment. On the other hand, urinary tract infections (UTIs) (2–41%) [10–14] and bloodstream infections (1.3–6%) [12–16] due to treatable pathogens were also documented.

Through a successful scale-up of malaria control interventions, Ethiopia has achieved remarkable reductions in disease burden, with declines in mortality and incidence of 96 and 89%, respectively, between 1990 and 2015 [17]. The most recent national malaria indicator survey in children under 5 found a 2-week period prevalence of fever of 16%, but only 0.6% prevalence of malaria [18], indicating the major role of non-malaria causes of fever. There have been recent investigations of bloodstream infections relating to *Salmonella* disease [19] and pneumonia [20] in febrile children in Ethiopia. However, comprehensive data on the relative contribution of various pathogens to acute febrile illness in Ethiopian children are lacking. Therefore, we aimed to describe common aetiologies of fever in children attending a tertiary hospital in southern Ethiopia, a setting where malaria transmission has declined. We also evaluated the susceptibility of bacterial isolates to commonly prescribed antimicrobials.

## Methods

### Study setting

Our prospective cross-sectional study was conducted at Hawassa University Comprehensive Specialized Hospital (HUCSH) in Ethiopia. Hawassa City is the capital of the Southern Nations and Nationalities Peoples’ Region (SNNPR) and had an estimated population of 455,658 (26.4% rural residents, 50% female) in 2017 [21]. The city is situated on the shore of Lake Awassa at 1708 m above sea level, with a temperature range of 9–29 °C [22], and a mean annual rain fall of 961 mm [23]. HUCSH is the largest tertiary hospital in the administrative region with 450 beds and provides medical services for the population in and beyond the region, allowing for the recruitment of children with severe febrile illnesses and thus associated pathogens.

Transmission of malaria in Ethiopia mainly occurs at elevations < 2000 m (m) above sea level, whereas areas with altitude > 2500 m above sea level are generally free of malaria. The incidence of malaria peaks during September to December, following the main rainy season (June – August), and there is a smaller transmission period from April to May [24]. In recent years, the number of districts with high malaria transmission in the country has significantly reduced, and moderate transmission has been reported in the study area [24]. Human immunodeficiency virus (HIV) prevalence among children aged 0–14 years in urban Ethiopia in 2017–2018 from population-based HIV impact assessment was 0.3% [25]. The *Haemophilus influenzae* type b vaccine, pneumococcal conjugate vaccine, and monovalent rotavirus vaccine were introduced into the national childhood immunization program in 2007, 2011, and 2013, respectively. The national coverage for full vaccination among children aged 12–23 months, as defined by the Ethiopian vaccination schedule, was 43% in 2019 [26].

### Patient enrolment

**Fig. 1:**
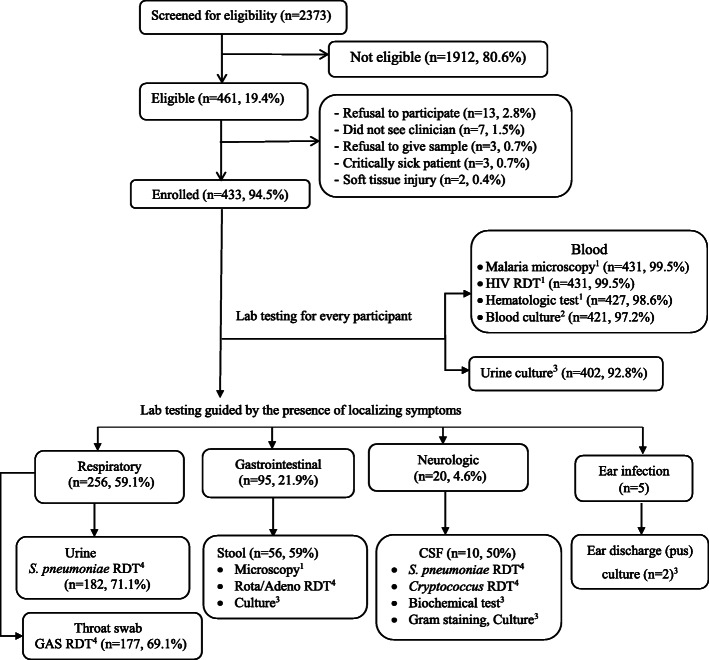
Participant screening, enrolment, and laboratory investigations at HUCSH, 2018–2019. RDT, rapid diagnostic test; CSF, cerebrospinal fluid; GAS, group A *Streptococcus.* ^1^ Routinely available test. ^2^ Test available sometimes, but only for hospitalized patients with clinical indications. ^3^ Test available commonly, but only for hospitalized patients with clinical indications. ^4^ Test made available by the study

As shown in Fig. 1, children who presented to the HUCSH paediatric outpatient department during regular working hours (8:00 AM – 5:00 PM) of each working day (Monday to Friday) were screened for eligibility and their caregivers offered enrolment of the children in the study during a 10-month period from May 2018 through February 2019. Eligible children were those aged at least 2 months and under 13 years with fever (axillary temperature of ≥37.5 °C or a history of fever episode at least once in the past 48 h), lasting no more than 7 days. Children requiring immediate lifesaving treatment were excluded if blood or urine cultures were not required as part of their care at admission. Patients whose main reason for the visit was injury, trauma, or skin and soft tissue infections were also excluded. Those children who had not met the eligibility criteria or whose caregivers declined to provide consent were advised that they could continue receiving the routine care service provided at the hospital.

### Sample size

A sample size of 440 was estimated using a single proportion formula, assuming prevalences of bacteraemia of 4.2, and 5.9% of UTIs based on a report from Tanzania [10], aiming for a 95% confidence level with 2.2% precision. Consecutive patients meeting inclusion criteria were enrolled until the sample size was achieved.

### Clinical and laboratory investigations

Nurses and doctors trained in study procedures gathered data from caregivers on the child’s demographic characteristics, history of any known chronic disease and vaccination status, treatment for the presenting fever episode prior to the visit, and presenting symptoms using a paper-based clinical case-report-form. Additionally, physical examination including vital signs and anthropometric measurements was done. As appropriate for each patient, clinicians requested the laboratory investigations routinely available in the hospital. As shown in Fig. 1, additional laboratory investigations implemented for this study were recorded on a paper-based laboratory case-report-form, and included examinations of stool for rotavirus/adenovirus, urine and cerebrospinal fluid (CSF) for *Streptococcus pneumoniae*, and throat swab for group A *Streptococcus* (GAS). Under normal hospital procedures, blood and urine cultures would have been available only for hospitalized patients with specific clinical indications.

#### Blood collection

Following standard procedures, a maximum of 7 ml venous blood (5 ml for blood culture, 2 ml EDTA blood for other tests) was drawn from children aged at least 5 years, and 5 ml of blood (3 ml for culture, 2 ml EDTA blood) was collected from children aged 2–59 months.

#### Aerobic blood culture

A single culture bottle inoculated with a blood sample was incubated in an automated BacT/ALERT 3D system (Biomerieux, France) for a maximum of 5 days. Blood cultures flagged as positive were Gram-stained and subcultured on MacConkey, chocolate, and blood agar plates (Oxide, UK) following standard microbiological techniques. Bacterial isolates were identified based on colony morphology, Gram reaction and biochemical reaction. Yeasts on Gram stained smears from positive blood cultures were identified based on morphology; no attempt was made to identify the fungal species. *Staphylococcus aureus* was differentiated from coagulase-negative staphylococci (CoNS) based on slide and tube coagulase test methods [27]. An isolate was considered a blood culture contaminant in instances when CoNS, viridans streptococci, *Micrococcus* species, *Bacillus* species, and *Corynebacterium* species were detected [14, 28].

#### Blood smear microscopy

Giemsa-stained thick and thin blood smear slides were examined by experienced microscopists to detect and identify blood parasites (*Plasmodium* species, *Borrelia* species, and other haemoparasites). A negative result was declared if no haemoparasite was seen after scanning a minimum of 200 consecutive microscopic fields in a thick blood smear.

#### HIV testing

Screening for HIV antibodies was performed according to the national algorithm using three RDTs in series. Each blood sample was screened using the Beijing Wantai HIV 1 + 2 Ab rapid test (Beijing Wantai Biological Pharmacy Enterprise Co., Ltd., China), and a negative test result was reported without the need for further confirmation. A positive screening test result was reported after confirmation with the HIV1/2 STAT-PAK assay (Chembio Diagnostic Systems Inc., USA). For discordant results, Unigold HIV (Trinity Biotech Plc., Ireland) was used as a tiebreaker. HIV seropositive samples from children aged 18 months or less were confirmed using polymerase chain reaction test.

#### Complete blood count

EDTA blood was analyzed using an automated haematology analyser (Shenzhen Mindray Biomedical Electronics Co., Ltd., China) for total and differential white blood cell count and haematocrit determination.

#### Urinalysis and culture

Study nurses assisted caregivers in collection of midstream urine samples from participants using a sterile container. Urine samples were also obtained from admitted patients who underwent urethral catheterization procedure as part of their medical care. Samples were analysed using dipstick and microscopy for early management according to routine practice in the hospital. Urine was also cultured on blood agar and MacConkey agar plates (Oxide, UK) to isolate bacterial pathogens using standard microbiological techniques. Urine culture showing significant bacteriuria (a single type of organism with ≥10^5^ and ≥ 10^4^ colony-forming-unit (CFU)/ml of urine collected by clean catch and urethral catheterization, respectively) was considered as indicative of urinary tract infection. Mixed culture was classified as urine contamination [27, 29].

#### Stool analysis and culture

A single stool specimen was collected from children whose caregivers reported diarrhoea/dysentery or if stool investigation was requested by attending clinicians. Stool samples were processed using direct microscopy (saline and iodine mounts) and modified Ziehl-Neelsen technique to detect intestinal protozoa, and subcultured on MacConkey and Xylose Lysine Deoxychocolate agar plates (Oxoid, UK) following enrichment in Selenite-F broth to isolate *Salmonella/Shigella* species. Identification of isolates was based on colony morphology, Gram reaction and biochemical reaction [27]. Samples were also tested using Rota/Adeno Antigen Rapid Test (Rapid Labs Ltd., UK).

#### Throat swab and urine RDTs

In participants with respiratory symptoms, a throat swab was collected and tested for GAS antigen using QuickVue In-Line Strep A test (Quidel Corporation, USA). Urine samples from these patients were tested using Alere BinaxNOW® *S. pneumoniae* Antigen Card (Alere Scarborough Inc., USA).

#### CSF and discharge analyses

As part of routine clinical care, CSF samples were collected from patients with suspected meningitis and routine analyses (Gram staining, cell count, protein and glucose measurements, and culture) were performed. For the purpose of this study, CSF samples from these patients were also screened for *S. pneumoniae* using Alere BinaxNOW® *S. pneumoniae* Antigen Card (Alere Scarborough Inc., USA) and *Cryptococcus* antigen using CrAg Lateral Flow Assay (IMMY, USA). As part of routine care, clinicians also collected ear swab samples in instances children had draining pus from ear, and bacterial cultures were performed following standard microbiological techniques [27].

#### Antimicrobial susceptibility testing

Antimicrobial susceptibility testing was performed using the Kirby-Bauer disc diffusion method [30] and interpreted according to the criteria of the Clinical and Laboratory Standards Institute (CLSI) [31]. Selected panels of antimicrobial discs that represent antimicrobials commonly prescribed in the study area and recommended by the CLSI guideline for each bacterial isolate were tested. Accordingly, antimicrobials included for testing were ampicillin (10 μg), amoxicillin and clavulanic acid (20/10 μg), cefoxitin (30 μg), ceftriaxone (30 μg), ceftazidime (30 μg), chloramphenicol (30 μg), ciprofloxacin (5 μg), clindamycin (10 μg), trimethoprim-sulfamethoxazole (1.25/23.75 μg), erythromycin (15 μg), gentamicin (10 μg), meropenem (10 μg), nitrofurantoin (300 μg), norfloxacin (10 μg), penicillin G (10 IU), and tetracycline (30 μg) (Oxoid, UK). Reference strains of *Escherichia coli* (ATCC – 25922), *Klebsiella pneumoniae* (ATCC – 700603), *Staphylococcus aureus* (ATCC – 25923), and *Pseudomonas aeruginosa* (ATCC – 27553) were tested as controls.

### Case definitions

Malaria was defined as positive blood smear microscopy for asexual stage of *Plasmodium* species [14]. Bloodstream infections (bacteraemia/fungaemia) was defined as positive blood culture for pathogenic bacteria/fungal cells [15]. Leucocytosis was defined as high total white blood cell count for age (age: 2–6 month (m), > 17,500 cells/μl; 7-24 m, > 17,000 cells/μl; 25-59 m, > 15,500 cells/μl; 5–8 year (y), > 14,500 cells/μl; 9-12y, > 13,500 cells/μl). Leukopenia was defined as low total white blood cell count for age (age: 2-24 m, < 6000 cells/μl; 25-59 m, < 5500 cells/μl; 5-8y, < 5000 cells/μl; 9-12y, < 4500 cells/μl). Anaemia was defined as low haematocrit value for age (age: 2 m, < 28%; 3-6 m, < 29%; 7-24 m, < 33%; 25 m-6 y, < 34%; 7-12y, < 35%) [32]. Tachypnea was defined as high respiratory rate for age (age: 2-11 m, ≥50 breaths/min; 12–59 m, ≥40 breaths/min; 5-12y, ≥30 breaths/min). Tachycardia was defined as high pulse rate for age (age: 2-11 m, > 160; 12-47 m, > 130 beats/min; 48 m-5y, > 120 beats/min; 6-8 y, > 115 beats/min; 9-12 y, > 110 beats/min) [33].

### Data analysis

Anthropometric z-scores were calculated using WHO AnthroPlus software. Categorical variables including demographic characteristics, clinical presentations, and laboratory findings were summarized using frequency and percentage. Duration of fever was expressed using median (interquartile range, IQR). Crude odds ratios (COR) were computed in bivariate logistic regression analysis for initial assessment of the association between laboratory findings and demographic and clinical characteristics. Adjusted odds ratios (AOR) were calculated in multivariable logistic regression analysis for variables that showed a significant association in bivariate analysis. A *p*-value < 0.05 was considered a significant association. Data were analysed using SPSS version-20 software.

## Results

### Enrolment and demographic characteristics

Of 2373 children screened for study eligibility (see Fig. 1), 1912 (80.6%) were not eligible because of their age, absence of fever, or fever that had already been present longer than 7 days. A total of 461 children met eligibility criteria, of whom 433 (93.9%) participated in this study. Reasons for non-participation were caregivers’ refusal (*n* = 13), departure from hospital before seeing the clinician (*n* = 7), refusal to provide a blood or urine specimen (*n* = 3), being critically ill (*n* = 3), or having a soft tissue injury (*n* = 2). Of 433 participants, 357 (82.4%) were under 5 years of age and 318 (73.4%) had completed vaccination. Severe wasting or stunting were found in 59 (13.7%) and 48 (11.1%) of 432 children, respectively (Table 1).

**Table 1:** Demographic, anthropometric, and vaccination status of febrile children attending HUCSH, 2018–2019

Characteristics	Frequency (%)
**Residence Adm. Region (*N*** **= 433)**	
SNNPR-Hawassa	258 (59.6)
SNNPR- other	60 (13.9)
Oromia	115 (26.6)
**Gender (*N*** **= 433)**	
Male	255 (58.9)
Female	178 (41.1)
**Age (*N*** **= 433)**	
2 to 11 m	132 (30.5)
12 to 35 m	148 (34.2)
36 to 59 m	78 (18.0)
5 to 7 y	39 (9.0)
8 to 12 y	36 (8.3)
**Vaccination status (*N*** **= 433)**	
Vaccinated for age	92 (21.2)
Completed	318 (73.4)
Other^b^	23 (5.3)
**Weight-for-age z-score (*N*** **= 419)**^**a**^	
Normal (≥ −2)	318 (75.9)
Moderate underweight (−3 to < − 2)	51 (12.2)
Severe underweight (< −3)	50 (11.9)
**Height-for-age z-score (*N*** **= 432)**	
Normal (≥ −2)	342 (79.2)
Moderate stunting (−3 to < −2)	42 (9.7)
Severe stunting (< −3)	48 (11.1)
**BMI-for-age z-score (*N*** **= 432)**	
Normal (≥ −2)	311 (72.0)
Moderate wasting (−3 to < −2)	62 (14.4)
Severe wasting (< −3)	59 (13.7)

*m* months, *y* years

*BMI* body-mass-index

^a^ Weight-for-age was calculated only for children up to 10 years of age

^b^Partially vaccinated (*n* = 9), unvaccinated (*n* = 11), unknown (*n* = 3)

### Clinical history and presentation

The median (IQR) duration of fever in the study participants was 3 (1–4) days, and 62 children (14.3%) had axillary temperature ≥ 39 °C. In addition to fever, the most common presenting clinical features (Table 2) were tachypnea, present in 244 children (56.4%), cough reported in 230 (53.1%), tachycardia recorded in 169 (39%), and vomiting recorded in 160 children (37%).

**Table 2:** Clinical history and presentation of children attending HUCSH, 2018–2019

Characteristics	Frequency (%) (***N*** = 433)
**Clinical history**	
Chronic disease^b^	16 (3.7)
Duration of fever	
1 day	118 (27.3)
2–4 days	239 (55.2)
5–7 days	76 (17.6)
**Most frequently reported symptoms**	
Cough	230 (53.1)
Vomiting	160 (37.0)
Fast breathing	79 (18.2)
Diarrhoea	82 (18.9)
Headache	24 (15.6)^a^
Sneezing /rhinorrhoea	46 (10.6)
Sore throat	16 (10.4)^a^
Grunting	33 (7.6)
Abdominal pain	28 (6.5)
Dysuria/urine frequency	9 (2.1)
Rash	9 (2.1)
**Vital signs**	
Axillary temperature	
< 37.5 °C	46 (10.6)
37.5–38.9 °C	325 (75.1)
≥ 39 °C	62 (14.3)
Tachypnea	244 (56.4)
Tachycardia	169 (39.0)
**Most frequently reported systemic signs**	
Throat	
Pharyngeal erythema	55 (12.7)
Tonsillar enlargement	48 (11.1)
Lung	
Retraction	78 (18.0)
Chest indrawing	35 (8.0)
Crepitation	106 (24.5)
Abdomen	
Abdominal tenderness	12 (2.8)
Hepatomegaly	22 (5.1)
Lymphadenopathy	12 (2.7)

^a^ Among children ≥3 years of age (*N* = 154)

^b^ Chronic disease reported by caregiver [heart disease (*n* = 9), asthma (*n* = 2), seizure disorder (*n* = 1), tuberculosis (*n* = 1), epilepsy (*n* = 1), paralysis of limps (*n* = 1), right mandibular swelling (*n* = 1)]

### Laboratory findings

Laboratory analyses detected one type of pathogen in 138 (31.9%) participants; two different pathogens in 26 (6%), and three in 3 (0.7%) of 433 children. None of the pathogens that we tested for could be detected in 266 (61.4%) of 433 participants.

As shown in Table 3, malaria was found in 14 (3.2%) of 431 children by blood smear microscopy; 8 (1.8%) were due to *Plasmodium falciparum* and 6 (1.4%) to *P. vivax*. No *Borrelia* species or other haemoparasites were found. Of 431 participants, 3 (0.7%) had HIV infection. Bloodstream infections (BSIs) were found in 27 (6.4%) of 421 participants by blood culture. Bacteria were isolated in 24 (5.7%) of 421 participants, and fungal cells (yeasts) were found in 3 (0.7%) children. *S. aureus* was the leading bacterial isolate, found in 16 (3.8%) of 421 children, followed by *Klebsiella* species, detected in 4 participants (1%). BSI was found in one child with malaria, but not in children with HIV.

**Table 3:** Laboratory findings in febrile children attending HUCSH, 2018–2019

Laboratory testing ^**c**^	Frequency (%)
**Blood counts (*N*** **= 427)**	
Leukopenia	41 (9.6)
Leukocytosis	68 (15.9)
Neutrophilia	62 (14.5)
Lymphocytosis	2 (0.5)
Anaemia	51 (11.9)
**Blood tests (*N*** **= 431)**	
Malaria	14 (3.2)
*Plasmodium falciparum*	8 (1.8)
*Plasmodium vivax*	6 (1.4)
HIV	3 (0.7)
**Blood culture (*N*** **= 421)**	27 (6.4)
*Staphylococcus aureus*	16 (3.8)
*Klebsiella* species	4 (1.0)
Other^a^	7 (1.6)
**Urine culture (*N*** **= 402)**	74 (18.4)
*Escherichia coli*	37 (9.2)
*Klebsiella* species	16 (4.0)
*Staphylococcus aureus*	5 (1.2)
Other^b^	18 (4.5)
**Stool testing (*N*** **= 56)**	
Microscopy	
*Giardia lamblia*	2 (3.6)
*Cryptosporidium* species	1 (1.8)
Rotavirus /Adenovirus RDT	
Rotavirus	14 (25)
Adenovirus	4 (7.1)
Culture	
*Salmonella* Paratyphi A	1 (1.8)
*Shigella dysenteriae*	1 (1.8)
**Respiratory infection tests**	
Throat swab (*N* = 177) GAS	28 (15.8)
Urine (*N* = 182) *Streptococcus pneumoniae*	31 (17.0)
**Other**	
CSF (*N* = 10) viridians streptococci	1 (10)
Ear discharge (*N* = 2) *E. coli*	1 (−)
CoNS	1 (−)

*GAS group A Streptococcus*, *CSF* cerebrospinal fluid, *CoNS* coagulase-negative staphylococci, *RDT* rapid diagnostic test

^a^*Haemophilus parainfluenzae* (*n* = 2), *Haemophilus influenzae* (*n* = 1), *Enterrococcus* species (*n* = 1), fungal cells (*n* = 3)

^b^*Proteus vulgaris* (*n* = 3), *Morganella morganii* (*n* = 3), *Enterococcus* species (*n* = 3), *Providencia rettgeri* (*n* = 2), *Pseudomonas* species (*n* = 2), *Citrobacter* species (*n* = 2), *Streptococcus pyogenes* (*n* = 1), *Staphyloccocus saprophyticus* (*n* = 1), *Streptococcus agalactiae* (*n* = 1)

^c^ The total number of children tested (N) varied as inadequate or no sample was obtained for some children

Urine cultures were positive in 74 (18.4%) of 402 participants, with *E. coli* and *Klebsiella* species being detected in 37 (9.2%) and 16 (4%) children, respectively. Among 56 children whose stool specimens were analysed, rotavirus was detected in 14 (25%) samples, and *Giardia lamblia* in 2 (3.6%) samples. Stool cultures were positive in 2 (3.6%) samples; one for *Salmonella* Paratyphi A (1.8%) and one for *Shigella dysenteriae* (1.8%). A throat swab test for GAS and urine test for *S. pneumoniae* were positive in 28 (15.8%) of 177 and 31 (17.0%) of 182 participants with respiratory symptoms, respectively. Of 10 CSF samples analysed, viridans streptococci was detected in one sample with pleocytosis (> 5 cells/μl; 18 cells/μl), elevated protein level (> 50 mg/dl; 219 mg/dl), and decreased glucose level (< 40 mg/dl; 2 mg/dl). No CSF sample was found positive for C*ryptococcus* antigen.

### Treatment prior to study recruitment

As reported by caregivers, 106 (24.5%) of 433 participants had taken antimicrobials and 6 (1.4%) reported taking antimalarial drugs for the current episode of illness prior to the present visit. Bacteraemia was found in 4 (3.8%) of 104 participants reported taking antimicrobials compared to 20 (6.3%) of 317 participants who had not taken antimicrobials (COR 0.59; 95% CI 0.20–1.78). Further, a UTI was detected in 17 (17.5%) of 97 participants reported taking antimicrobials compared to 57 (18.7%) of 305 participants who reported not taking antimicrobials (COR 0.93; 95% CI 0.51–1.68).

### Association between the presence of infections and participants’ characteristics

Detailed findings are summarized in Supplementary Tables 1, 2 and 3. In multivariable logistic regression analysis, the odds of having malaria were significantly lower in children with axillary temperature < 39 °C compared to those with temperature **≥** 39.0 °C (AOR 0.23; 95% CI (0.07–0.75). Further, children aged 5 years and older were more likely to have malaria than those under 5 years of age (AOR 3.21; 95% CI 1.04–9.92) (Supplementary Table 1). Bloodstream infections were observed exclusively among children aged < 5 years (Supplementary Table 2). The odds of having a UTI was significantly higher in children aged under 36 months, and highest among those aged 2–11 months (AOR 4.99; 95% CI 1.96 – 12.7) compared to those aged 36–59 months. A UTI was more frequently detected among children with fever of 5–7 days duration compared to those with fever of 1 day (AOR 2.55; 95% CI 1.19–5.48), and among those with tachycardia (AOR 2.70; 95% CI 1.51–4.81) (Supplementary Table 3).

### Antimicrobial susceptibility

Antimicrobial susceptibility testing showed that 68 (97.1%) of 70 isolates from urine specimens were susceptible to nitrofurantoin and 58 (79.5%) of 73 were susceptible to norfloxacin (Table 4). Gram-positive and Gram-negative isolates from any sample were most consistently susceptible to ciprofloxacin. Of 21 isolates of *S. aureus*, 18 (85.7%) were susceptible to chloramphenicol, and 17 (81.9%) were susceptible to clindamycin. However, 68 (90.7%) of 75 isolates were resistant to ampicillin, 78 (82.1%) of 95 were resistant to trimethoprim-sulfamethoxazole, 71 (74%) of 96 were resistant to tetracycline, and 43 (59.7%) of 72 were resistant to amoxicillin and clavulanic acid.

**Table 4:** Antimicrobial susceptibility pattern of bacteria isolated from various samples in febrile children attending HUCSH, 2018–2019

Name of antimicrobial	*E. coli* n (%) (*N* = 38)		*Klebsiella* spp. n (%) (*N* = 20)		Other Gram-negative n (%) (*N* = 15)		*S. aureus* n (%) (*N* = 21)		Other Gram-positive (n/N)		All isolates n (%)^¶^	
	Susceptible	Resistant	Susceptible	Resistant	Susceptible	Resistant	Susceptible	Resistant	Susceptible	Resistant	Susceptible	Resistant
Ampicillin	0 (0)	37 (97.4)	1 (5.3)^b^	18 (90.0)^b^	4 (28.6)^d^	10 (71.4)^d^	–	–	(1/4)	(3/4)	6 (8.0)^m^	68 (90.7)^m^
Trimethoprim-sulfamethoxazole	3 (7.9)	35 (92.1)	3 (15.0)	17 (85.0)	4 (26.7)	11 (73.3)	7 (33.3)	14 (66.7)	(0/1)	(1/1)	17 (17.9)^p^	78 (82.1)^p^
Tetracycline	6 (15.8)	30 (78.9)	5 (25.0)	14 (70.0)	5 (35.7)^d^	7 (50.0)^d^	3 (15.0)^k^	16 (80.0)^k^	(0/4)	(4/4)	19 (19.8)^q^	71 (74.0)^q^
Amoxicillin and clavulanic acid	8 (21.1)	26 (68.4)	4 (20.0)	10 (50.0)	6 (42.9)^d^	7 (50.0)^d^	–	–	–	–	18 (25.0)^r^	43 (59.7)^r^
Ceftriaxone	14 (36.8)	20 (52.6)	7 (35.0)	13 (65.0)	7 (53.8)^e^	4 (30.8)^e^	–	–	(0/1)	(1/1)	28 (38.9)^r^	38 (52.8)^r^
Ceftazidime	15 (39.5)	21 (55.3)	5 (25.0)	13 (65.0)	9 (60.0)	5 (33.3)	–	–	–	–	29 (39.7)^t^	39 (53.4)^t^
Gentamicin	23 (60.5)	14 (36.8)	8 (40.0)	12 (60.0)	7 (63.6)^f^	4 (36.4)^f^	15 (71.4)	5 (23.8)	(4/5)	(1/5)	57 (60.0)^p^	36 (37.9)^p^
Chloramphenicol	23 (60.5)	15 (39.5)	9 (45.0)	11 (55.0)	9 (64.3)^d^	2 (14.3)^d^	18 (85.7)	3 (14.3)	(2/2)	0	61 (64.2)^p^	31 (32.6)^p^
Ciprofloxacin	26 (68.4)	8 (21.1)	15 (75.0)	1 (5.0)	13 (86.7)	1 (6.7)	16 (76.2)	4 (19.0)	(3/4)	0	73 (74.5)^u^	14 (14.3)^u^
Meropenem	33 (86.8)	2 (5.3)	16 (80.0)	1 (5.0)	7 (46.7)	2 (13.3)	–	–	–	–	56 (76.7)^t^	5 (6.8)^t^
Nitrofurantoin*	37 (100)^a^	0 (0)	15 (93.8)^c^	0 (0)	7 (87.5)^g^	1 (12.5)^g^	5 (100)^h^	0 (0)	(4/4)	0	68 (97.1)^w^	1 (1.4)^w^
Norfloxacin*	27 (73.0)^a^	9 (24.3)^a^	14 (87.5)^c^	1 (5.0)^c^	10 (90.9)^f^	1 (9.1)^f^	4 (80.0)^h^	1 (20.0)^h^	(3/4)	(1/4)	58 (79.5)^t^	13 (17.8)^t^
Penicillin G	–		–		–		3 (15.0)^k^	17 (85.0)^k^	(2/5)	(3/5)	5 (20.0)^x^	20 (80.0)^x^
Erythromycin	–		–		–		10 (47.6)	11 (52.4)	(1/2)	(1/2)	11 (47.8)^y^	12 (52.2)^y^
Cefoxitin	–		–		–		13 (61.9)	7 (33.3)	(1/1)	0	14 (63.6)^z^	7 (31.8)^z^
Clindamycin	–		–		–		17 (81.9)	4 (19.0)	(1/2)	(1/2)	18 (78.3)^y^	5 (21.7)^y^

*Tested for urine isolates as recommended by the guideline

Number of given isolates tested for the antimicrobials: *E.coli*: ^a^(*N* = 37); *Klebsiella* species: ^b^(*N* = 19), ^c^(*N* = 16); Other Gram-negative bacteria: ^d^(*N* = 14), ^e^(*N* = 13), ^f^(N = 11), ^g^(*N* = 8); *S. aureus*: ^h^(*N* = 5), ^k^(*N* = 20)

^¶^ Each type of isolate was tested for subsets of antimicrobials as recommended by the guideline; there are few missing cases: ^m^(*N* = 75), ^p^(*N* = 95), ^q^(*N* = 96), ^r^(*N* = 72) ^t^(*N* = 73), ^u^(*N* = 98), ^w^(*N* = 70), ^x^(*N* = 25), ^y^(*N* = 23), ^z^(*N* = 22)

## Discussion

To our knowledge, our study is the first systematic investigation of common aetiologies of acute febrile illness among children in Ethiopia, and one of only a handful of such studies from Africa. The findings showed that proportions of children with malaria, bloodstream infections, and urinary tract infections were 3.2, 6.4 and 18.4%, respectively.

Malaria was uncommon (3.2%) among febrile children. This finding is consistent with the substantial malaria reductions recorded in Ethiopia [18, 34], and associated with large-scale implementation of malaria control interventions, including the utilization of insecticide-treated mosquito nets, indoor residual spraying, and early diagnosis and treatment [24]. A further contributor to the low prevalence of malaria may be effective management in lower level health care, leading to few cases appearing at the tertiary hospital. Our finding was similar to that reported from a study in Kenya (5.2%) [35] although a higher (49.7%) result has been reported from a recent study in Burkina Faso [13]. The more frequent detection of malaria in children aged 5 years and above was consistent with findings in Gabon [36] and Tanzania [37] which have both seen a decreased malaria burden and shift in risk towards children older than 5 years. Consistent to a report elsewhere [35], clinical presentations other than a higher fever were not shown to be associated with malaria, reflecting that malaria can be difficult to diagnose clinically. A decline in malaria burden emphasizes the need for improving diagnostics to reliably rule out bacteraemia in febrile children and avoid inappropriate antimicrobial use.

Our study showed that 6.4% of febrile children had bloodstream infections. Similar results were found in investigations involving participants from referral hospitals in Tanzania (5.8%) [15] and Burkina Faso (6%) [13], and contrast with findings on bloodstream infection from lower level health facilities in Ethiopia (1.6%) [38] and Tanzania (1.7–3.2%) [12, 14]. Dominant blood culture isolates in the current study were *S. aureus* and *Klebsiella* species, while other studies that recruited participants from outpatient settings as well documented *Salmonella* Typhi (0.7–0.9%) [10, 12, 14], invasive non-typhoidal serovars of *Salmonella enterica* (4.5%) [13], and *S. pneumoniae* (0.2–0.5%) [12–14]. In Ethiopia, a low prevalence of salmonellosis (0.2%) in children was also reported recently during the Typhoid Fever Surveillance in Africa Program [38]. While culture-based diagnosis is the gold standard for diagnosing bacteraemia, and allows for testing antimicrobial susceptibility, it is unlikely to be feasible on a routine basis in resource-constrained settings [39]. The observed low proportion of blood cultures positive for a pathogen in febrile children attending outpatient department may point out blood culture testing services should be prioritised for patients with higher likelihood of bacteraemia including under-5 year old children with severe diseases.

The importance of UTI as cause of febrile illness among children is commonly overlooked in resource-constrained settings, due to non-specific symptoms in children and lack of availability of diagnostic tools. The proportion of urine culture positive cases in the current study (18.4%) was similar to that reported from a study in Tanzania (17.7%) [14] although both lower (2–5.6%) [11–13] and higher (41%) [10] results have been reported in other African settings. Difference in composition of enrolled participants in terms of clinical characteristics and local risk factors may have played a role in the observed disparity of results. The predominance of *E. coli* and *Klebsiella* species as the detected uropathogens was consistent with findings from other research in Africa [12, 14]. Untreated UTI can lead to serious renal disease [40], so better approaches for UTI evaluation in febrile children are needed. As shown in other studies [29, 41], we have found that UTI was more common in children aged under 3 years and those with longer duration of fever, emphasising the need for screening these groups with available tests such as urine dipstick and microscopy to ensure early management.

Among children with gastrointestinal symptoms, we detected *S. dysenteriae* (1.8%) and *Salmonella* Paratyphi A (1.8%) by stool culture. *S. flexneri* (20%), and *Salmonella* Typhi (1.9%) have been reported elsewhere [10]. The proportion of rotavirus infection detected (25%) was virtually identical to the findings of a recent analysis focussing on prevalence of rotavirus infection in children under-five in Ethiopia [42]. The occurrence of rotavirus infections in children aged under 3 years indicates the need to target this age group with rotavirus/adenovirus screening via RDT, to minimize antimicrobial over-prescription.

The burden of GAS in children has not been well investigated in Ethiopia, despite post-streptococcal immunological complications such as acute rheumatic fever, rheumatic heart disease, and glomerulonephritis being common [43]. The observed proportion of GAS (15.8%) in the present study was similar to that found by culture in children aged 5–15 years with pharyngitis in southwest Ethiopia (11.3%) [44]. Our finding underlines the importance of early diagnosis and prompt antimicrobial intervention in children with clinical indications to minimize long-term sequelae.

We found that the bacterial isolates were resistant to drugs such as ampicillin, trimethoprim-sulfamethoxazole, tetracycline, and amoxicillin and clavulanic acid, consistent with findings reported recently from Ethiopia [45, 46] and elsewhere [14]. Misuse and overuse of these drugs in relation to empiric treatment, prophylaxis, and self-medication may be contributing substantially to the development of resistance. In agreement with a recent report in the study area [46], most isolates from urine were susceptible to nitrofurantoin and norfloxacin. The first line empiric treatment for UTI based on the national guideline is trimethoprim-sulfamethoxazole [33] for which a high level of resistance by *E. coli* and other uropathogens was observed in our study. Further, our findings indicate that fluoroquinolones which are currently reserved as second line options for treating UTI in Ethiopia present a viable alternative as first line therapies.

Our study had a number of limitations. We only recruited for this study during weekday working hours, which may have led to some form of selection bias. In addition, we were limited in the breadth of diagnostic tests and pathogens that we were able to test for. Specifically, investigations for zoonotic bacterial infections, arboviruses, and respiratory viruses were not included, but would have been useful to inform fever management guidelines. Another limitation is in regard to the extent of detection we could achieve with a single blood culture, and the absence of samples for some children. RDTs were used for the diagnosis of some infections despite being not the gold standard diagnostic, so cases might have been over- or under-detected. Antimicrobials taken prior to enrolment might have resulted in prevalence of bacterial infections being under-estimated. Finally, we did not include any testing of non-febrile controls, limiting our ability to interpret the role of detected pathogens in contributing to the fever episode. Despite these limitations, our study has the strength of being the first in Ethiopia to assess a wide range of pathogens including bacteria, parasites, viruses, and fungi, within the same cohort of children. The enrolment of study participants over a 10-month period minimized the risk of missing infections predominating in certain seasons. The inclusion of children who were managed as both outpatients and inpatients may help understand pathogens involving in various clinical conditions.

## Conclusion

The study showed that malaria and bacteraemia were uncommon among febrile children presenting to the outpatient department of this tertiary hospital in Ethiopia. Febrile children presenting to lower level health facilities may have a different pathogen profile. The observed high proportion of UTI demand assessment of clinical algorithms to ensure prompt antibiotic intervention. The observed resistance to commonly used antimicrobials calls for stronger measures to ensure rational use of antimicrobial agents and reducing emergence and spread of drug resistant pathogens. Thus, improving access to diagnostics for appropriate management of specific pathogens, periodic investigations of aetiological agents, and assessing local antimicrobial susceptibility pattern are critically essential. Evidence from this study may be used to inform decision makers and health workers when planning and implementing measures to improve diagnostics, clinical management, and prevention of febrile diseases.

## Supplementary Information

**Additional file 1: Supplementary Table 1.** Distribution of malaria by demographic and clinical characteristics of febrile children attending HUCSH, 2018–2019.

**Additional file 2: Supplementary Table 2.** Distribution of bloodstream infections by demographic and clinical characteristics of febrile children attending HUCSH, 2018–2019.

**Additional file 3: Supplementary Table 3.** Distribution of urinary tract infection by demographic and clinical characteristics of febrile children attending HUCSH, 2018–2019.

## Data Availability

The datasets used and/or analysed during the current study are available from the corresponding author on reasonable request.

## Abbreviations

RDT: Rapid diagnostic test
WHO: World Health Organization
UTI: Urinary tract infection
HUCSH: Hawassa University Comprehensive Specialized Hospital
SNNPR: Southern Nations and Nationalities Peoples’ Region
HIV: Human immunodeficiency virus
CSF: Cerebrospinal fluid
GAS: Group A *Streptococcus*
CoNS: Coagulase-negative staphylococci
CFU: Colony-forming-unit
CLSI: Clinical and Laboratory Standards Institute
IQR: Interquartile range
COR: Crude odds ratio
AOR: Adjusted odds ratio
BSIs: Bloodstream infections

## References

[1] Reiner RC, Olsen HE, Ikeda CT, Echko MM, Ballestreros KE, Manguerra H (2019). Diseases, injuries, and risk factors in child and adolescent health, 1990 to 2017: findings from the global burden of diseases, injuries, and risk factors 2017 study. JAMA Pediatr.

[2] World Health Organization. Children: reducing mortality 2017. Available from: https://www.who.int/news-room/fact-sheets/detail/children-reducing-mortality. Accessed 02 May 2020.

[3] World Health Organization (2013). WHO informal consultation on fever management in peripheral health care settings: a global review of evidence and practice.

[4] Crump JA, Kirk MD (2015). Estimating the burden of febrile illnesses. PLoS Negl Trop Dis.

[5] Crump JA (2014). Time for a comprehensive approach to the syndrome of fever in the tropics. Trans R Soc Trop Med Hyg.

[6] Masaninga F, Sekeseke-Chinyama M, Malambo T, Moonga H, Babaniyi O, Counihan H (2012). Finding parasites and finding challenges: improved diagnostic access and trends in reported malaria and anti-malarial drug use in Livingstone district, Zambia. Malar J.

[7] World Health Organization. Malaria prevention works: let's close the gap Geneva, Siwezerland 2017. Available from: http://apps.who.int/iris/bitstream/10665/254991/1/WHO-HTM-GMP-2017.6-eng.pdf?ua=1. Accessed 16 Jan 2018.

[8] Medernach RL, Logan LK (2018). The growing threat of antibiotic resistance in children. Infect Dis Clin N Am.

[9] Zaman S, Hussain M, Nye R, Mehta V, Mamun TK, Hossain N (2017). A review on antibiotic resistance: alarm bells are ringing. Cureus.

[10] D'Acremont V, Kilowoko M, Kyungu E, Philipina S, Sangu W, Kahama-Maro J (2014). Beyond malaria causes of fever in outpatient Tanzanian children. N Engl J Med.

[11] Elfving K, Shakely D, Andersson M, Baltzell K, Ali AS, Bachelard M (2016). Acute uncomplicated febrile illness in children aged 2-59 months in Zanzibar - Aetiologies, antibiotic treatment and outcome. PLoS One.

[12] Hildenwall H, Amos B, Mtove G, Muro F, Cederlund K, Reyburn H (2016). Causes of non-malarial febrile illness in outpatients in Tanzania. Tropical Med Int Health.

[13] Kiemde F, Tahita MC, Lompo P, Rouamba T, Some AM, Tinto H (2018). Treatable causes of fever among children under five years in a seasonal malaria transmission area in Burkina Faso. Infect Dis Poverty.

[14] Mahende C, Ngasala B, Lusingu J, Butichi A, Lushino P, Lemnge M (2014). Aetiology of acute febrile episodes in children attending Korogwe District hospital in North-Eastern Tanzania. PLoS One.

[15] Crump JA, Ramadhani HO, Morrissey AB, Msuya LJ, Yang LY, Chow SC (2011). Invasive bacterial and fungal infections among hospitalized HIV-infected and HIV-uninfected children and infants in northern Tanzania. Tropical Med Int Health.

[16] D'Acremont V, Malila A, Swai N, Tillya R, Kahama-Maro J, Lengeler C (2010). Withholding antimalarials in febrile children who have a negative result for a rapid diagnostic test. Clin Infect Dis.

[17] Deribew A, Dejene T, Kebede B, Tessema GA, Melaku YA, Misganaw A (2017). Incidence, prevalence and mortality rates of malaria in Ethiopia from 1990 to 2015: analysis of the global burden of diseases 2015. Malar J.

[18] Ethiopian Public Health Institute. Ethiopia National Malaria Indicator Survey 2015. Addis Ababa: Ethiopian Public Health Institute; 2016. https://www.ephi.gov.et/images/pictures/download2009/MIS-2015-Final-Report-December-_2016.pdf. Accessed 16 Jan 2018.

[19] Marks F, von Kalckreuth V, Aaby P, Adu-Sarkodie Y, El Tayeb MA, Ali M (2017). Incidence of invasive salmonella disease in sub-Saharan Africa: a multicentre population-based surveillance study. Lancet Glob Health.

[20] Negash AA, Asrat D, Abebe W, Hailemariam T, Hailu T, Aseffa A (2019). Bacteremic community-acquired pneumonia in Ethiopian children: Etiology, antibiotic resistance, risk factors, and clinical outcome. Open Forum Infect Dis.

[21] Agency FDRoECS. Population projection of Ethiopia for all regions at *Wereda* level from 2014–2017. Addis Ababa, Ethiopia2013.

[22] Kasim FO, Abshare MW, Mukuna TE, Wahab B (2018). Land use and ambient air quality in Bahir Dar and Hawassa, Ethiopia. Air Soil Water Res.

[23] Belete MD, Diekkrüger B, Roehrig J (2017). Linkage between water level dynamics and climate variability: the case of Lake Hawassa hydrology and ENSO phenomena. Climate..

[24] Taffese HS, Hemming-Schroeder E, Koepfli C, Tesfaye G, Lee MC, Kazura J (2018). Malaria epidemiology and interventions in Ethiopia from 2001 to 2016. Infect Dis Poverty..

[25] Federal Ministry of Health. Ethiopia population-based HIV impact assessment 2017-2018. https://phia.icap.columbia.edu/wpcontent/uploads/2020/02/EPHIA-Summary-Sheet_ARV-adjusted_Feb2020.pdf. Accessed 16 Oct 2020.

[26] Ethiopian Public Health Institute and ICF. Ethiopia Mini Demographic and Health Survay 2019: Key indicators. Rockville: EPHI and ICF.

[27] Cheesbrough M. District laboratory practice in tropical countries. Part 2. 2nd ed. New York: Cambridge University Press; 2006.

[28] Hall KK, Lyman JA (2006). Updated review of blood culture contamination. Clin Microbiol Rev.

[29] Robinson JL, Finlay JC, Lang ME, Bortolussi R (2014). Urinary tract infections in infants and children: diagnosis and management. Paediatr Child Health.

[30] Bauer AW, Kirby WM, Sherris JC, Turck M (1966). Antibiotic susceptibility testing by a standardized single disk method. Am J Clin Pathol.

[31] Clinical and Laboratory Standards Institute. Performance Standards for Antimicrobial Susceptibility Testing. CLSI supplement M100S 26th ed. Wayne: Clinical and Laboratory Standards Institute, Pennsylvania USA 2016.

[32] Nathan DG, Oski FA. Hematology of infancy and childhood 2 e, editor. Philadelphia: W.B. Saunders; 1981.

[33] Federal Ministry of Health (2016). Pediatric hospital care: Ethiopia. Pocket Book. Guidelines of the managment of common illnesses in hospitals.

[34] Girum T, Shumbej T, Shewangizaw M (2019). Burden of malaria in Ethiopia, 2000-2016: findings from the Global Health estimates 2016. Trop Dis Travel Med Vaccines.

[35] O'Meara WP, Mott JA, Laktabai J, Wamburu K, Fields B, Armstrong J (2015). Etiology of pediatric fever in western Kenya: a case-control study of falciparum malaria, respiratory viruses, and streptococcal pharyngitis. Am J Trop Med Hyg.

[36] Mawili-Mboumba DP, Akotet MKB, Kendjo E, Nzamba J, Medang MO, Mbina J-RM (2013). Increase in malaria prevalence and age of at risk population in different areas of Gabon. Malar J.

[37] Winskill P, Rowland M, Mtove G, Malima RC, Kirby MJ (2011). Malaria risk factors in north-East Tanzania. Malar J.

[38] Teferi M, Desta M, Yeshitela B, Beyene T, Cruz Espinoza LM, Im J (2019). Acute febrile illness among children in Butajira, South-Central Ethiopia during the typhoid fever surveillance in Africa Program. Clin Infect Dis.

[39] Opota O, Croxatto A, Prod'hom G, Greub G (2015). Blood culture-based diagnosis of bacteraemia: state of the art. Clin Microbiol Infect.

[40] Roberts KB, Subcommittee on Urinary Tract Infection, Steering Committee on Quality Improvement and Management (2011). Urinary tract infection: clinical practice guideline for the diagnosis and management of the initial UTI in febrile infants and children 2 to 24 months. Pediatrics..

[41] White B (2011). Diagnosis and treatment of urinary tract infections in children. Am Fam Physician.

[42] Damtie D, Melku M, Tessema B, Vlasova AN (2020). Prevalence and genetic diversity of rotaviruses among under-five children in Ethiopia: A systematic review and meta-analysis. Viruses..

[43] Nigussie B, Tadele H (2019). Heart failure in Ethiopian children: mirroring the unmet cardiac services. Ethiop J Health Sci.

[44] Tesfaw G, Kibru G, Mekonnen D, Abdissa A (2015). Prevalence of group a β-haemolytic Streptococcus among children with pharyngitis in Jimma town, Southwest Ethiopia. Egypt J Ear Nose Throat Allied Sci.

[45] Ibrahim RA, Teshal AM, Dinku SF, Abera NA, Negeri AA, Desta FG (2018). Antimicrobial resistance surveillance in Ethiopia: Implementation experiences and lessons learned. Afr J Lab Med..

[46] Mitiku E, Amsalu A, Tadesse BT (2018). Pediatric urinary tract infection as a cause of outpatient clinic visits in southern Ethiopia: a cross-sectional study. Ethiop J Health Sci.

