# Supplementary material for: Aetiology of acute febrile illness among children attending a tertiary hospital in southern Ethiopia
Source: BMC Infect Dis. 2020 Nov 30;20:903. doi: 10.1186/s12879-020-05635-x (PMC7706267; doi:10.1186/s12879-020-05635-x)
Supplement: Supplementary file 1 — Additional file 1: Supplementary Table 1. Distribution of malaria by demographic and clinical characteristics of febrile children attending HUCSH, 2018–2019. [file 12879_2020_5635_MOESM1_ESM.docx]

Supplementary Table 1: Distribution of malaria by demographic and clinical characteristics of febrile children attending HUCSH, 2018-2019

| **Characteristics** | **Malaria** | | | |
| --- | --- | --- | --- | --- |
|  | **n (%)**  **blood tested (N=431)** | **n (%)^ᴥ^ positive** | **COR (95% CI)** | **AOR (95% CI)** |
| **Gender** |  |  |  |  |
| Male | 253 (58.7) | 10 (4.0) | 1.79 (0.55-5.80) | - |
| Female | 178 (41.3) | 4 (2.2) | 1 |  |
| **Age** |  |  |  |  |
| 2-59 m | 356 (82.6) | 8 (2.2) | 1 | 1 |
| ≥5 y | 75 (17.4) | 6 (8.0) | 3.78 (1.27-11.2)* | 3.21 (1.04-9.92)* |
| **Sneezing/rhinorrhoea** |  |  |  |  |
| Yes | 46 (10.7) | 0 | - | - |
| No | 385 (89.3) | 14 (3.6) |  |  |
| **Cough** |  |  |  |  |
| Yes | 230 (53.4) | 3 (1.3) | 0.23 (0.06-0.83)* | 0.27 (0.07-1.02) |
| No | 201 (46.6) | 11 (5.5) | 1 | 1 |
| **Diarrhoea** |  |  |  |  |
| Yes | 81 (18.8) | 1 (1.2) | 0.32 (0.04-2.51) | - |
| No | 350 (81.2) | 13 (3.7) | 1 |  |
| **Vomiting** |  |  |  |  |
| Yes | 159 (36.9) | 6 (3.8) | 1.29 (0.44-3.80) | - |
| No | 272 (63.1) | 8 (2.9) | 1 |  |
| **Headache** |  |  |  |  |
| Yes | 24 (15.7)^a^ | 4 (16.7) | 3.49 (0.94.-13.0) | - |
| No | 129 (84.3)^a^ | 7 (5.4) | 1 |  |
| **Axillary temperature** |  |  |  |  |
| <37.5 ^0^C | 46 (10.7) | 1 (2.2) | 0.20 (0.02-1.76) | 0.19 (0.02-1.70) |
| 37.5-38.9 ^0^C | 324 (75.2) | 7 (2.2) | 0.20 (0.07-0.63)* | 0.23 (0.07-0.75)* |
| ≥39 ^0^C | 61 (14.2) | 6 (9.8) | 1 | 1 |
| **Tachypnea** |  |  |  |  |
| Yes | 243 (56.4) | 8 (3.3) | 1.03 (0.35-3.03) | - |
| No | 188 (43.6) | 6 (3.2) | 1 |  |
| **WBC count** |  |  |  |  |
| Normal | 318 (74.5)^b^ | 12 (3.8) | 1 | - |
| High | 68 (15.9)^b^ | 1 (1.5) | 0.38 (0.05-2.98) |  |
| Low | 41 (9.6)^b^ | 1 (2.4) | 0.64 (0.08-5.03) |  |
| **Weight-for-age z-score** |  |  |  |  |
| Normal (≥ -2) | 317 (76.0)^c^ | 12 (3.8) | 1 | - |
| Underweight (< -2) | 100 (24.0)^c^ | 1 (1.0) | 0.26 (0.03-2.99) |  |
| **Height-for-age z-score** |  |  |  |  |
| Normal (≥ -2) | 341 (79.3)^d^ | 12 (3.5) | 1 | - |
| Stunting (< -2) | 89 (20.7)^d^ | 2 (2.2) | 0.63 (0.14-2.87) |  |
| **BMI-for-age z-score** |  |  |  |  |
| Normal (≥ -2) | 310 (72.1)^d^ | 11 (3.5) | 1 | - |
| Wasting (< -2) | 120 (27.9)^d^ | 3 (2.5) | 0.70 (0.19-2.54) |  |

m, months; y, years

* Significantly associated (p-value < 0.05)

COR, crude odds ratio; AOR, adjusted odds ratio; CI, confidence interval; WBC, white blood cell; BMI, body-mass-index

**^ᴥ^** Percentages within categories of the characteristics

^a^(N=153), ^b^(N=427),  ^c^(N=417), ^d^(N=430)
