# Supplementary material for: Aetiology of acute febrile illness among children attending a tertiary hospital in southern Ethiopia
Source: BMC Infect Dis. 2020 Nov 30;20:903. doi: 10.1186/s12879-020-05635-x (PMC7706267; doi:10.1186/s12879-020-05635-x)
Supplement: Supplementary file 2 — Additional file 2: Supplementary Table 2. Distribution of bloodstream infections by demographic and clinical characteristics of febrile children attending HUCSH, 2018–2019. [file 12879_2020_5635_MOESM2_ESM.docx]

Supplementary Table 2: Distribution of bloodstream infections by demographic and clinical characteristics of febrile children attending HUCSH, 2018-2019

| Characteristics | Bloodstream infections | | | |
| --- | --- | --- | --- | --- |
|  | **n (%)**  **blood cultured**  **(N=421)** | **n (%)^ᴥ^ positive** | **COR (95% CI)** | **AOR (95% CI)** |
| Gender |  |  |  |  |
| Male | 247 (58.7) | 17 (6.9) | 1.21 (0.54-2.72) | - |
| Female | 174 (41.3) | 10 (5.7) | 1 |  |
| Age |  |  |  |  |
| 2-11 m | 124 (29.5) | 13 (10.5) | 2.14 (0.67-6.81) | - |
| 12-35 m | 145 (34.4) | 10 (6.9) | 1.35 (0.41-4.46) |  |
| 36-59 m | 77 (18.3) | 4 (5.2) | 1 |  |
| ≥5 y | 75 (17.8) | 0 | - |  |
| Sneezing/rhinorrhoea |  |  |  |  |
| Yes | 46 (10.9) | 5 (10.9) | 1.96 (0.70-5.45) | - |
| No | 375 (89.1) | 22 (5.9) | 1 |  |
| Cough |  |  |  |  |
| Yes | 225 (53.4) | 20 (8.9) | 2.63 (1.09-6.37)* | 1.70 (0.60-4.76) |
| No | 196 (46.6) | 7 (3.6) | 1 | 1 |
| Diarrhoea |  |  |  |  |
| Yes | 79 (18.8) | 8 (10.1) | 1.92 (0.81-4.55) | - |
| No | 342 (81.2) | 19 (5.6) | 1 |  |
| Vomiting |  |  |  |  |
| Yes | 156 (37.1) | 14 (9.0) | 1.91 (0.87-4.18) | - |
| No | 265 (62.9) | 13 (4.9) | 1 |  |
| Axillary temperature |  |  |  |  |
| <37.5 ^0^C | 43 (10.2) | 1 (2.3) | 1 | - |
| 37.5-38.9 ^0^C | 317 (75.3) | 22 (6.9) | 3.13 (0.41-23.8) |  |
| ≥39 ^0^C | 61 (14.5) | 4 (6.6) | 2.95 (0.32-27.3) |  |
| Tachypnea |  |  |  |  |
| Yes | 238 (56.5) | 20 (8.4) | 2.31 (0.95-5.58) | - |
| No | 183 (43.5) | 7 (3.8) | 1 |  |
| Crepitation |  |  |  |  |
| Yes | 102 (24.2) | 12 (11.8) | 2.70 (1.22-5.89)* | 1.99 (0.79-5.0) |
| No | 319 (75.8) | 15 (4.7) | 1 | 1 |
| Retraction |  |  |  |  |
| Yes | 76 (18.1) | 7 (9.2) | 1.65 (0.67-4.05) | - |
| No | 345 (81.9) | 20 (5.8) | 1 |  |
| Known chronic disease |  |  |  |  |
| Yes | 16 (3.8) | 3 (18.8) | 3.66 (0.98-13.7) | - |
| No | 405 (96.2) | 24 (5.9) | 1 |  |
| WBC count |  |  |  |  |
| Normal | 312 (74.6)^e^ | 20 (6.4) | 1 | - |
| High | 66 (15.8)^e^ | 6 (9.1) | 1.46 (0.56-3.79) |  |
| Low | 40 (9.6)^e^ | 1 (2.5) | 0.37 (0.05-3.87) |  |
| Weight-for-age z-score |  |  |  |  |
| Normal (≥ -2) | 310 (76.2)^f^ | 17 (5.5) | 1 | - |
| Underweight (< -2) | 97 (23.8)^f^ | 10 (10.3) | 1.98 (0.88-4.48) |  |
| Height-for-age z-score |  |  |  |  |
| Normal (≥ -2) | 334 (79.5)^g^ | 17 (5.1) | 1 | 1 |
| Stunting (< -2) | 86 (20.5)^g^ | 10 (11.6) | 2.45 (1.08-5.57)* | 2.18 (0.94-5.04) |
| BMI-for-age z-score |  |  |  |  |
| Normal (≥ -2) | 302 (71.9)^g^ | 18 (6.0) | 1 | - |
| Wasting (<-2) | 118 (28.1)^g^ | 9 (7.6) | 1.30 (0.57-2.99) |  |

m, months; y, years

* Significantly associated (p-value < 0.05)

COR, crude odds ratio; AOR, adjusted odds ratio; CI, confidence interval; WBC, white blood cell; BMI, body-mass-index

**^ᴥ^** Percentages within categories of the characteristics

^e^(N=418), ^f^(N=407),  ^g^(N=420)
