# Supplementary material for: Aetiology of acute febrile illness among children attending a tertiary hospital in southern Ethiopia
Source: BMC Infect Dis. 2020 Nov 30;20:903. doi: 10.1186/s12879-020-05635-x (PMC7706267; doi:10.1186/s12879-020-05635-x)
Supplement: Supplementary file 3 — Additional file 3: Supplementary Table 3. Distribution of urinary tract infection by demographic and clinical characteristics of febrile children attending HUCSH, 2018–2019. [file 12879_2020_5635_MOESM3_ESM.docx]

Supplementary Table 3: Distribution of urinary tract infection by demographic and clinical characteristics of febrile children attending HUCSH, 2018-2019

| Characteristics | n (%) urine cultured (N=402) | n (%)^ᴥ^ positive | COR (95% CI) | AOR (95% CI) |
| --- | --- | --- | --- | --- |
| Gender |  |  |  |  |
| Male | 241 (60) | 43 (17.8) | 1 | - |
| Female | 161 (40) | 31 (19.3) | 1.10 (0.66-1.83) |  |
| Age |  |  |  |  |
| 2-11 m | 112 (27.9) | 31 (27.7) | 3.83 (1.59-9.23)* | 4.99 (1.96-12.7)* |
| 12-35 m | 138 (34.3) | 27 (19.6) | 2.43 (1.01-5.89)* | 2.33 (0.94-5.77) |
| 36-59 m | 77 (19.2) | 7 (9.1) | 1 | 1 |
| ≥5 y | 75 (18.7) | 9 (12.0) | 1.36 (0.48-3.87) | 1.30 (0.45-3.79) |
| Duration of fever |  |  |  |  |
| 1 day | 111 (27.6) | 17 (15.3) | 1 | 1 |
| 2-4 days | 221 (55) | 36 (16.3) | 1.08 (0.57-2.02) | 0.99 (0.52-1.89) |
| 5-7 days | 70 (17.4) | 21 (30.0) | 2.37 (1.15-4.90)* | 2.55 (1.19-5.48)* |
| Cough |  |  |  |  |
| Yes | 213 (53) | 45 (21.1) | 1.48 (0.88-2.47) | - |
| No | 189 (47) | 29 (15.3) | 1 |  |
| Diarrhoea |  |  |  |  |
| Yes | 74 (18.4) | 20 (27.0) | 1.88 (1.04-3.39)* | 1.41 (0.75-2.66) |
| No | 328 (81.6) | 55 (16.5) | 1 | 1 |
| Vomiting |  |  |  |  |
| Yes | 149 (37.1) | 30 (20.1) | 1.20 (0.72-2.01) | - |
| No | 253 (62.9) | 44 (17.4) | 1 |  |
| Axillary temperature |  |  |  |  |
| <37.5 ^0^C | 45 (11.2) | 4 (8.9) | 1 | - |
| 37.5-38.9 ^0^C | 300 (74.6) | 62 (20.7) | 2.67 (0.92-7.74) |  |
| ≥39 ^0^C | 57 (14.2) | 8 (14.0) | 1.67 (0.47-5.96) |  |
| Tachypnea |  |  |  |  |
| Yes | 228 (56.7) | 45 (19.7) | 0.82 (0.49-1.38) | - |
| No | 174 (43.3) | 29 (16.7) | 1 |  |
| Tachycardia |  |  |  |  |
| Yes | 164 (40.8) | 39 (23.8) | 1.81 (1.09-3.01)* | 2.70 (1.51-4.81)* |
| No | 238 (59.2) | 35 (14.7) | 1 | 1 |
| Dysuria/urine frequency |  |  |  |  |
| Yes | 9 (2.2) | 1 (11.1) | 0.55 (0.07-4.45) | - |
| No | 393 (97.8) | 73 (18.6) | 1 |  |
| Chronic disease |  |  |  |  |
| Yes | 15 (3.7) | 3 (20) | 1.11 (0.31-4.05) | - |
| No | 387 (96.3) | 71 (18.3) | 1 |  |
| Abdominal pain |  |  |  |  |
| Yes | 26 (6.5) | 2 (7.7) | 0.35 (0.08-1.52) | - |
| No | 376 (93.5) | 72 (19.1) | 1 |  |
| WBC count |  |  |  |  |
| Normal | 295 (74.1)^k^ | 55 (18.6) | 1 | - |
| High | 65 (16.3)^k^ | 11 (16.9) | 0.89 (0.44-1.81) |  |
| Low | 38 (9.5)^k^ | 8 (21.1) | 1.16 (0.51-2.68) |  |

m, months; y, years

*Significantly associated (p-value < 0.05)

COR, crude odds ratio; AOR, adjusted odds ratio; CI, confidence interval; WBC, white blood cell; BMI, body-mass-index

**^ᴥ^** Percentages within categories of the characteristics

^k^(N=398)
